# A Child as a Donor for Hematopoietic Stem Cell Transplantation: Bioethical Justification—A Case Study on Sickle Cell Disease

**DOI:** 10.1155/2017/8394732

**Published:** 2017-02-23

**Authors:** Andrea Z. Pereira, Ricardo Hellman, Nelson Hamerschlak, Andrea Kondo, Polianna Mara Rodrigues de Souza, Wilson Leite Pedreira, Luiz Fernando Alves Lima Mantovani, Eduardo Juan Troster, Henrique Grunspun, Marco Aurélio Scarpinella Bueno

**Affiliations:** ^1^Oncology, Hematology, and Bioethical Committee, Hospital Israelita Albert Einstein, São Paulo, SP, Brazil; ^2^Oncology and Hematology, Hospital Israelita Albert Einstein, São Paulo, SP, Brazil; ^3^Hematology and Bone Marrow Transplantation, Hospital Israelita Albert Einstein, São Paulo, SP, Brazil; ^4^Bioethical Committee, Hospital Israelita Albert Einstein, São Paulo, SP, Brazil

## Abstract

Hematopoietic stem cell transplantation (HSCT) is an important treatment option for children with severe and refractory sickle cell disease (SCD) with debilitating clinical complications. HSCT with cells from the bone marrow of a HLA-identical sibling used in SCD has a low mortality risk, high cure rate, and high event-free survival rate after a median follow-up of 5-6 years. However, matched donors are found in only about 20% of the patients. A boy aged 8 years with SCD had a sister, <2 years old, a fully compatible donor. The boy met all eligibility criteria to undergo HSCT, and he was suffering from cognitive and neurologic impairment due to ischemic events. A Bioethical Committee jointly discussed the ethical issues on this case after a pediatric evaluation released the very young sister for donation. The justification was that the sister would benefit from the donation too because of the greater likelihood of survival and cure and less suffering of her brother. The parents were informed about the risks and benefits for both children, and the family was psychologically evaluated. After their consent, HSCT was performed and the patient is cured from SCD. The complication for the donor was the need for blood transfusion.

## 1. Introduction

Sickle cell disease (SCD) is a severe inherited disease which affects multiple organs in the body, causing acute and chronic complications, with significant morbidity and reduced life expectancy [1–3]. Hematopoietic stem cell transplantation (HSCT) is an important treatment option for children with severe and refractory disease and debilitating clinical complications [3–5]. Moreover, in the last 10 years, HSCT has been considered the only definitive curative treatment for severe SCD, with small mortality and morbidity rates [1, 3, 6–12]. Until 2014, about 500 SCD children had undergone HSCT in the world, resulting in a SCD-free survival rate of 95% [11]. The benefits of HSCT for SCD include family quality of life, stabilization, or restoration function in affected organs (central nervous system and lung) [6, 7].

Gene therapy might be an option for SCD cure, mainly for patients who do not have HLA-identical relative donors, but the treatment is still in the early stages of testing [7, 8, 10].

HSCT with cells from the bone marrow of a HLA-identical sibling used in SCD has a low mortality risk (5–10%), high cure rate (90%), and high event-free survival rate after a median follow-up of 5-6 years (85–100%) [2, 6, 13]. However, matched donors are found in only about 20% of the patients [13]. For this reason, considering the bioethical point of view, HSCT in severe SCD has been a valuable therapeutic option [6]. In Brazil, only related HSCT is allowed for SCD.

The objective of this study is to present a bioethical dilemma in the case of a young boy undergoing HSCT for SCD.

## 2. Case Report

Our patient is a SCD patient, a boy aged 8 years, living in São Paulo, Brazil. He met all eligibility criteria to undergo HSCT (Table 1) [1, 2, 7, 8].

He had an ischemic stroke, the major cause of morbidity in SCD [14], at 8 months of age, without motor or sensory sequelae, showing only mild cognitive impairment. He has been under treatment with monthly blood transfusions since the ischemic event. As a complication of SCD, he exhibited a splenic sequestration crisis and underwent splenectomy at the age of three years.

After seven years of monthly transfusion program, he presented an iron overload, with heart disease and mild diastolic congestive heart failure. Even though these blood transfusions have prevented neurologic events, they have brought about significant end-organ toxicity in this patient. This was expected [8], since generally after 2 years regular blood transfusion therapy causes overload of iron side effects [14, 15].

The clinical and laboratory features supported HSCT indication, and the patient has an HLA-matched sister (10 × 10). Postponing his transplant could lead to increased cardiac involvement, which would increase the morbidity of BMT.

His sister, a girl aged 1 year and 5 months, was identified as a possible donor. She was evaluated by two different pediatricians, one from the Hematology Department at Hospital Israelita Albert Einstein and the second pediatrician from another hospital, the Child Cancer Institute (Hospital das Clínicas, Faculdade de Medicina da Universidade de São Paulo, USP). After these evaluations, the sister was released for donation.

For HSCT, bone marrow donation may be performed by surgical harvesting of the bone marrow or by apheresis collection. The patient's surgical harvesting has the benefit of lower risk of graft-versus-host disease (GVHD) after transplantation [16].

To perform the surgical harvesting, it would be necessary to collect a volume equivalent to 15 ml/kg of the recipient patient, in order to achieve the sufficient amount of progenitor cells, minimizing the risk of graft failure. In the literature, there is a recommendation that each collection does not exceed the maximum volume of 20 ml/kg of the donor [17, 18].

In this case, the patient weighs 26 kg and his sister weighs 9 kg. Thus, it would be necessary for the procedure for bone marrow donation to be carried out 2 or 3 times, in order to reach the sufficient quantity of cells to HSCT [17, 18].

Bone marrow collection could be performed under general anesthesia through multiple punctures to the posterior iliac crest, with low risk of severe adverse events [14, 19, 20].

As this donor is compatible with her brother (here reported) and her sister, both diagnosed with sickle cell anemia and indicated a bone marrow transplant, we decided to collect the 20 ml/kg donor volume to maintain a fraction stored for future transplantation of sister, reducing the need for additional collections.

These considerations led to the consultation of the Bioethical Committee of the hospital as follows.

## 3. The Bioethical Problem and the Bioethical Committee

The reason for the consultation of the bioethics committee was the ethical conflict that their parents would be subjected to, on consenting to the procedures, due to their difficulty in understanding their son and daughter's risks and benefits.

For the hematologists, it could be very difficult to decide about the risks involved in bone marrow donation because of the young age of the donor: the girl is only 1 year and 5 months old. She was the only possible donor for the patient; however, to do so, she would be submitted to three bone marrow surgical harvesting procedures, in order to obtain enough material for her brother.

The HSCT Team requested an ethics consultation with the Bioethical Committee (BC) to obtain necessary help on deciding what to do about the donor age and how to support family decision [21]. The BC is made up of physicians from different medical specialties. However, the following specialists were invited to discuss the case: three hematologists, three pediatricians, one oncology psychologist, the Intensive Care Unit physician coordinator, and the children anesthesiologist, all of them members of the staff of the Hospital Israelita Albert Einstein staff. The argumentation was based on risks and benefits to the donor.

At the time of this specialist meeting, the sister donor had already been evaluated by two pediatricians of another hospital, who were in favor of the procedure. Their parents had already signed the informed consent after a meeting with HSCT Team, and all of the donation risks had been explained.

The considerations presented to them were as follows: unfortunately, studies showed the most unfavorable results and more complications with unrelated donors for HSCT in SCD, so the patient's sister was in fact the best option [8, 12]. Generally, HLA-matched siblings are considered to be the best donors for HSCT [21, 22], but only approximately 14% of SCD patients have matched sibling donors available [12]. It is very common that in HSCT performed in children donors are children too (about 30%) [22].

Some studies have shown psychological benefits to donors, such as increased self-esteem, pride in donating, a greater sensitivity to the needs of others, increased family union, feeling like a better person, and an increased meaning and worth of life [22–25]. Specifically for HSCT donors, positive and negative outcomes were established; however these studies evaluated adolescents and children who were older than the donor in this case [24–26].

Although some studies have shown that HSCT donation is safe, with only temporary and modest discomfort (1 severe adverse event/453 donations), there is a higher risk of needing a blood transfusion, fatigue, wound infection, and pain. Also, cardiovascular complications can occur after general anesthesia is used for catheter insertion (stem cell harvest) or for bone marrow collection from the hip [23, 27, 28]. The donors in the highest risk for complications were less than four years old, as in the case of our donor [28]. In 1987, for instance, a brother, who was 19 months old and HLA-compatible, donated his bone marrow to his older sister. He did not have any complication related to the donation and, at the time of the publication, he was 26 years old. This boy's birth had been planned genetically for the donation, but despite this and after much discussion, the case was considered ethical after many discussions [29].

The risk of death from HSCT donation is considered low: 1/10.000 donations [25]. In our Brazilian Hospital, after more than 10 years of HSCT procedures, no donor died.

Our decision for the donation was based on the recommended criteria bellow [25, 27, 28, 30]:

1. Hospital Israelita Albert Einstein is a HSCT referral center;
2. The donor would have a multidisciplinary pediatrician team to do the prior transplant evaluation and the systematic follow-up after that;
3. The donor and her family would have psychological evaluation and support prior to and after to transplant;
4. Donor risk would not be significant, based on scientific studies and clinical practice in our hospital;
5. Without the transplantation, there was a severe risk of death and complications in our SCD patient at that time. The boy could not wait for his sister to get older;
6. There were not any medically equivalent histocompatible adult donors among relatives who would be able to donate;
7. There is a strong and positive emotional relationship between the donor and the recipient;
8. There was a reasonable probability that the recipient would benefit from the HSCT;
9. Clinical emotional and psychosocial risks to the donor would be reduced;
10. Parental permission consent was obtained after the parents had been informed of the risks and the benefits of the procedure.

## 4. Follow-Up

After three months of BC decision, the first bone marrow donation was carried out by surgical harvesting of the bone marrow. The donor had to receive a blood transfusion, and this was the only donation complication. Despite the fact that the hematology team programmed three donation procedures, only two donations were really performed, because the volume of the bone marrow collected was enough.

The first bone marrow collection was performed on November 30, 2015, with a volume of 191 ml and total nucleated cells of 1.34 × 10^8^/kg and CD34 positive cells of 4.42 × 10^6^/kg.

We choose to cryopreserve the product and perform a new collection in order to reach the minimum dose of nucleated cells, reducing the risk of failure of primary grafting.

The second collection was performed on February 23, 2016, after a reevaluation of the donor, which verified complete recovery after the first collection. The collected volume was 173 ml, with total nucleated cells of 1.97 × 10^8^/kg and CD34 cells of 5.06 × 10^6^/kg. The donor did not have any significant side effects and she is still healthy until now.

The HSCT was performed without significant complications and our patient has been cured from SCD. Four months after his HSCT, he had 85% of donor-host hematopoietic chimerism, which is sufficient to overcome a genetic defect [5].

## 5. Discussion

Our BC discussion was based on the “four principles approach to medical ethics”: autonomy, beneficence, nonmaleficence, and justice [31, 32].

Firstly, we discussed the risk-benefits for the procedure with the multidisciplinary team, in a BC meeting, based on the nonmaleficence principle [31, 33]. We concluded that this principle would be respected in this case, as the benefits were larger than the risks for the donor. Studies about HSCT donation in pediatrics considered it an ethical issue, based on the* primum non nocere* principle, because it is an altruistic and solidary practice, without a lot of risks for donors [33].

Although studies have shown that iron overload caused by regular transfusions can be controlled by an oral iron chelator [34], our patient had already have severe blood transfusion consequences. Furthermore, these problems could more probably reduce survival and quality of life than the HSCT procedure itself. Therefore, the* beneficence* and the* nonmaleficence* principles were respected in this point of view [34].

Parents should protect and represent their children, and, in the case of HSCT sibling donation, they must be assisted by health care providers to decide about it [30]. In some studies, autonomy, another bioethical principle, is not considered a real problem in the case of a HSCT minor donor, because the decision is validated by parents [33, 35]. In our case, BC concluded that the informed consent form (IC) was explained adequately by the HSCT Team, using language easy to understand, discussing the benefits and risks of donation and HSCT, and answering the parents' doubts [36]. Even though the donor herself could not possibly have signed the informed consent form, because of her young age and lack of comprehension, her parents were secure about their decision and signed it being aware of the important information.

The justice bioethical principle, the fair distribution of goods and services [35], in HSCT, refers to the favorable assessment of financial resources available [33]. Even though this family was unable to pay for the HSCT procedure and health insurance, they were financed by a SCD Governmental Program, which would pay for all the expenses in a private Brazilian Hospital. Therefore, the justice principle has been respected in this case.

An important factor to consider in such cases is also the competent support from relatives and/or caregivers during the pre- and post-HSCT period [34]. In our case, these parents and/or caregivers would be able to support the donor and the recipient (their daughter and son). BC investigated it and had guaranteed family conditions to give the necessary support to their kids.

Finally, our patient, his donor, and their parents were able to comprehend the procedure, and they had the educational, cultural, family, and social background according to the “rule of descending order.” This rule suggests that patients have less knowledge than the physician, justifying our efforts to inform them adequately [34].

## 6. Conclusion

The accepted justification for permitting a minor sibling to donate bone marrow stem cells was that the donor, the sister, would benefit from it because of the greater likelihood of survival and cure and less suffering for her brother. After discussing all essential points with the multidisciplinary team, the BC considered that all important conditions were satisfied in order to provide more benefits and fewer risks to the HSCT patient and his donor. Thus, the donation and HSCT were recommended by the BC.

## Figures and Tables

**Table 1:** Eligibility criteria for hematopoietic stem cell transplantation (HSCT) in sickle cell disease (SCD) [7].

Essential condition	Availability of a matched sibling donor
Eligibility criteria	Stroke
	Elevated transcranial Doppler velocity
	Venoocclusive episodes
	Pulmonary hypertension
	Tricuspid regurgitation jet velocity > 2,5 ms
	Osteonecrosis and/or avascular necrosis
	Red cell alloimmunization
	Silent stroke especially with cognitive impairment
	Recurrent priapism
	Sickle nephropathy

## Abbreviations

HSCT: Hematopoietic stem cell transplantation
SCD: Sickle cell disease
GVHD: Graft-versus-host Disease
BC: Bioethical Committee.
